# Robotic microscopy for everyone: the OpenFlexure microscope

**DOI:** 10.1364/BOE.385729

**Published:** 2020-04-08

**Authors:** Joel T. Collins, Joe Knapper, Julian Stirling, Joram Mduda, Catherine Mkindi, Valeriana Mayagaya, Grace A. Mwakajinga, Paul T. Nyakyi, Valerian L. Sanga, Dave Carbery, Leah White, Sara Dale, Zhen Jieh Lim, Jeremy J. Baumberg, Pietro Cicuta, Samuel McDermott, Boyko Vodenicharski, Richard Bowman

**Affiliations:** 1 Centre for Photonics and Photonic Materials, Department of Physics, University of Bath, UK; 2 Ifakara Health Institute, Ifakara, Tanzania; 3 STICLab, Dar Es Salaam, Tanzania; 4 Department of Chemistry, University of Bath, UK; 5 Centre for Nanoscience and Nanotechnology, Department of Physics, University of Bath, UK; 6 Nanophotonics Centre, Cavendish Laboratory, University of Cambridge, UK; 7 Cavendish Laboratory, University of Cambridge, UK

## Abstract

Optical microscopes are an essential tool for both the detection of disease in clinics, and for scientific analysis. However, in much of the world access to high-performance microscopy is limited by both the upfront cost and maintenance cost of the equipment. Here we present an open-source, 3D-printed, and fully-automated laboratory microscope, with motorised sample positioning and focus control. The microscope is highly customisable, with a number of options readily available including trans- and epi- illumination, polarisation contrast imaging, and epi-florescence imaging. The OpenFlexure microscope has been designed to enable low-volume manufacturing and maintenance by local personnel, vastly increasing accessibility. We have produced over 100 microscopes in Tanzania and Kenya for educational, scientific, and clinical applications, demonstrating that local manufacturing can be a viable alternative to international supply chains that can often be costly, slow, and unreliable.

## 1. Introduction

For centuries optical microscopy has been the foundation of scientific imaging and analysis in many aspects of medicine, life, and physical sciences [1]. Commercially available high-end microscopes can provide precise motorised high-resolution imaging capabilities, however these are often prohibitively expensive. This is compounded in the developing world where unreliable supply chains inflate prices, and slow acquisition [2]. Maintaining equipment adds further burden as devices have often not been designed for these operating environments [3], and neither manufacturers representatives nor spare parts are available locally, leading to much of the laboratory equipment in resource-poor countries being out-of-service [4].

Open-source hardware is poised to revolutionise the distribution of scientific instrumentation, impacting research, local manufacturing, and education [5,6]. Open science hardware designs are already in use for both research and education across a wide range of disciplines [7–11]. Within research laboratories in particular, 3D printers have become an increasingly common item of equipment. This has led a number of open science hardware projects to use 3D printing as an accessible platform for locally prototyping and manufacturing laboratory-grade devices [10–12].

Here we present the OpenFlexure Microscope (OFM), a 3D-printed, laboratory-grade automated microscope. The OFM aims to make automated microscopy accessible both to projects with a modest budget in established labs, and to developing nations aiming to adopt more advanced scientific research technologies. Unlike other low-cost open-source microscopes the OFM has a strong focus on providing precise 3D motion for focus and sample positioning, built around a well-characterised flexure mechanism [13]. This mechanism provides 3-axis positioning with step sizes as low as 50 nm in the z axis, and 70 nm in x and y. The range of mechanical motion is smaller than traditional mechanical stages, with 12×12×4 mm travel, and due to the primarily plastic construction, our design has a limited load capacity unsuitable for very large or heavy samples. However, both the range of motion, and load capacity, are ample for most microscopy applications. The microscope is both compact, fitting within a 15cm×15cm×20cm volume, and lightweight at ≈ 500 g in its highest resolution, fully automated configuration. The microscope is designed to be highly modular, and while the positioning mechanism is standard across all configurations, illumination, optics, and imaging can be completely customized. A range of included interchangeable optics modules enable different imaging modalities (Section 2.1) allowing different cameras and imaging lenses to be used for applications from blood smear parasitology to school teaching.

**Fig. 1.**
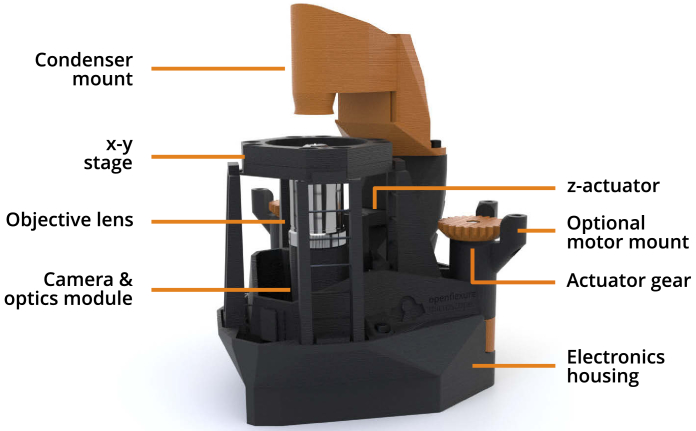
Overview of the OpenFlexure Microscope design, in transmission bright-field configuration. The condenser mount houses an illumination LED and a plastic condenser lens, while the optics module sits below the stage and houses an objective lens, tube lens, and camera. The entire optics module is attached to the z-actuator, providing variable focus. Both the optics module and x-y stage are controlled by actuator gears at the back of the microscope, optionally driven by stepper motors. A detachable electronics housing stores optional electronic parts, such as motor controllers and a Raspberry Pi, for automation.

The OFM has now been trialled in a range of applications, and reproduced by a number of groups around the world. The design (Fig. 1) has been refined to reduce assembly time, ease component sourcing, and maximise reliability. These ongoing optimisations are key to a sustainable and useful open hardware project that is trusted for critical applications. Version 6.0 of the OpenFlexure Microscope represents a well tested microscopy platform, enabling the rapid prototyping of new instrumentation, and replication of high quality research tools, even in resource-poor settings.

## 2. Imaging capabilities

The OpenFlexure Microscope uses an interchangeable optics module to easily change between imaging modes. Most of the optics modules use standard RMS objectives to yield high-quality images expected from a laboratory microscope, and an option is available to use an inverted, spatially offset webcam lens to provide magnification at a low-cost for educational use. The estimated parts costs for various configurations of the microscope are provided in Appendix A.1.

We employ an 8MP CMOS sensor (Raspberry Pi camera V2) to provide digital imaging capabilities. The front lens of the camera is easily unscrewed and removed, allowing the optics module to properly function while still providing high-quality imaging. Many low-cost microscopy projects make use of smartphone cameras for imaging [14–16], however as these retain the smartphone lens as part of the optical path they usually require additional costly optical components. In contrast, by using a well characterized, widely available sensor (Sony IMX219) [17], the OFM is able to reliably reproduce the reported imaging quality at minimal cost. Furthermore, a permanent, low-cost imaging sensor makes long-running automated experiments significantly more practical, as discussed in section 2.3. A comprehensive radiometric characterization of the IMX219 sensor has quantified the shot, read, and fixed-pattern noise of the sensor, its spectral response, and its stability, demonstrating the Raspberry Pi camera’s suitability for scientific imaging [18].

The high-quality optics module makes use of achromatic objectives and an achromatic tube lens to minimize chromatic aberrations. While narrow-band illumination sources would help further minimize chromatic aberrations, full-color white light imaging is typically required for our applications such as blood parasite imaging (section 2.1.1) and graphene flake imaging (section 2.1.2). Nevertheless, by introducing achromatic optics, the dominant chromatic effect becomes the chief ray angle color mixing, which can be largely mitigated with software calibration [19].

**Fig. 2.**
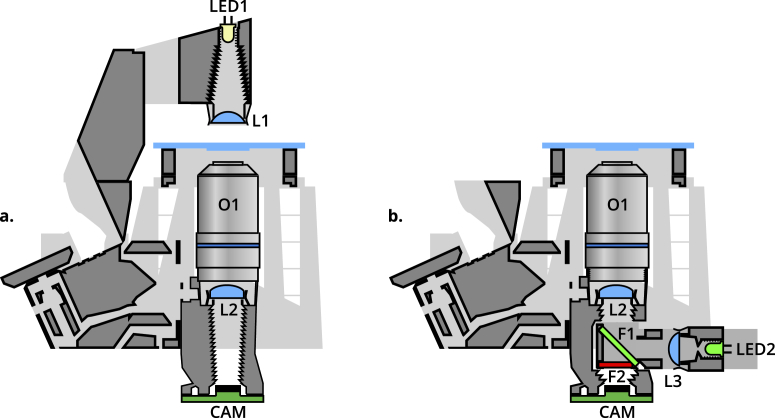
Cross-section schematics of trans- (**a.**) and epi- (**b.**) illumination configurations. LED1 provides transmission illumination, with a condenser lens L1. LED2 and lens L3 provide epi-illumination, connected to a removable filter cube housing two filters, F1 at 45° and F2. These filters can be removed or replaced by beamsplitters or polarizers to enable bright-field, fluorescence, or polarization-contrast epi-illuminated imaging. In both configurations, a standard RMS objective O1 and tube lens L2 image the sample onto the camera sensor CAM.

OFM optics modules are available for bright-field (with both trans- and epi- illumination) imaging, polarisation contrast imaging, and epifluorescence imaging. Additionally, both finite and infinite conjugate objectives can be used by varying the position of the tube lens. Figure 2 provides schematics of trans- (a.) and epi- (b.) illumination configurations. Fluorescence imaging is realised by inserting a dichroic filter in position F1, and a long-pass filter in position F2 of the removable filter cube. The cube is connected to a reflection illuminator (L3, LED2) with suitable LED illumination. Bright-field epi- imaging is achieved by removing filter F2, and using a standard beamsplitter in position F1. Polarisation contrast imaging requires transmission illumination (Fig. 2(a)), with a linear polariser in filter cube position F2.

**Fig. 3.**
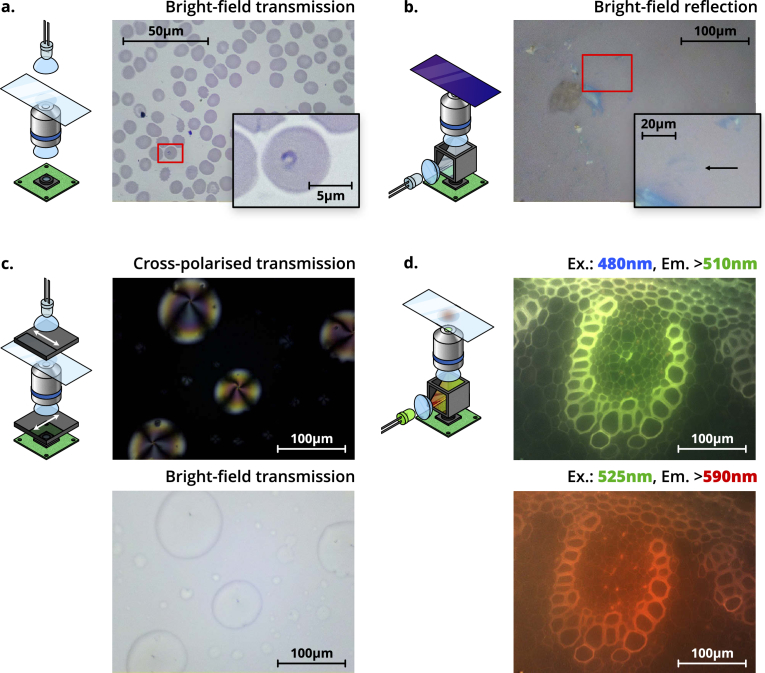
Schematics (left) and images (right) of four different imaging modalities possible using the OFM. **a.** Trans-illumination bright-field imaging of a Giemsa-stained thin blood smear, obtained with a 100×, 1.25NA oil immersion objective. The inset shows a magnified section of the image, highlighting a ring-form trophozoite of *Plasmodium falciparum*. **b.** Epi-illumination bright-field imaging of a group of thin graphene flakes, obtained with a 40×, 0.65NA dry objective. The inset shows a magnified section of the image, highlighting a resolvable tri-layer graphene flake (contrast has been digitally enhanced for clarity by increasing brightness and gamma). **c.** Polarisation-contrast trans-illumination image of 5CB liquid crystal droplets. A bright-field image is shown below for comparison. Both images were obtained with a 40×, 0.65NA dry objective, and depict the same region of the sample. Arrows on the polarisers denote the transmission axis. **d.** Fluorescence images of unstained Lily of the Valley (*convallaria majalis*) rhizome, at two different wavelengths. The excitation wavelength (“Ex.”), and minimum emission wavelength imaged (“Em.”) are shown above the respective panels. Both images were obtained with a 40×, 0.65NA dry objective, and depict the same region of the sample. Both the colour of illumination LED, and the filters in the filter cube, will change depending on the application.

Images in this section are obtained with an RMS objective (100×, 1.25NA oil immersion, or 40×, 0.65NA dry), an f=50 mm tube lens (ThorLabs AC127-050-A), and an 8MP CMOS sensor (Raspberry Pi camera V2) (Fig. 3(a)).

### 2.1 Imaging modes

#### 2.1.1 Bright-field trans-illumination

Bright-field trans-illumination is the “standard” imaging mode for the OFM. White light illumination, provided by an LED, is focused onto the sample using an f=5 mm PMMA condenser lens. LED illumination is diffused by sanding the LED case, preventing the condenser from imaging the chip onto a sample. Figure 3(a) shows an image of a Giemsa-stained blood smear, obtained at the Ifakara Health Institute for the purpose of malaria diagnosis. The image is obtained with a 100×, 1.25NA oil immersion objective and a 16.25 ms acquisition time. The microscope was vibrationally isolated with a layer of foam below the device. In this configuration, individual red blood cells, with dimensions on the order of microns, are clearly resolved. A ring-form trophozoite of *Plasmodium falciparum* within a red blood cell is highlighted in the figure inset (contrast has been digitally enhanced for clarity by increasing brightness and gamma). Despite being only 1 µm to 2 µm across, the morphology of these ring-form parasites is also clearly resolved by the OFM.

Image quality is in line with expectations for manual microscopes currently used for malaria diagnosis. However, digital images make it possible to re-examine a slide without physically preserving and transporting it. This improves record-keeping, quality control, and training of both new and experienced technicians. Automatic acquisition of images (Section 2.3) goes further, improving throughput of samples and paving the way for automated image processing to assist diagnostic technicians.

#### 2.1.2 Bright-field epi-illumination

Reflection (epi-) bright-field imaging is enabled by inserting a 50/50 beam splitter at 45° within a printed filter cube between the tube lens and the sensor. Illumination is provided by a collimated diffused LED reflected through the objective by the beamsplitter (Fig. 2(b)). Light reflected from the sample is partially transmitted through the beam splitter and onto the sensor, enabling imaging of opaque samples.

Figure 3(b) shows graphene flakes on a 300 nm SiO2/Si substrate, illuminated with a white LED, and imaged using a 40×, 0.65NA dry objective with a 9 ms acquisition time. This provides enough resolving power to image typical graphene flakes, while simplifying use by removing the need for immersion oil. Illumination is provided by a Nichia NSPL500DS white LED, with ≈20 µW power at the sample plane. Graphene flakes, produced via mechanical exfoliation, are widely used to study novel electrical and mechanical properties of 2D materials [20]. Thin graphene flakes, with random positions and thicknesses across the substrate, are commonly identified by manual optical microscopy. Subtle variations in the colour and intensity of transmitted light (≈2.3% absorption per layer [20,21]) can be used to estimate their thickness. This method of identifying flakes is both time consuming and subjective when performed manually, but can be automated with the OFM. Raman spectroscopy measurements have confirmed that the flake highlighted in Fig. 3(b) is tri-layer graphene. Unlike other methods of identifying flakes (AFM, Raman spectroscopy, etc.), this searching method is high speed and low-cost, and the graphene is not damaged. By optimising illumination for the detection of graphene flakes [22,23], and improving image processing [20], we estimate the OFM can resolve even monolayer graphene, and we have also imaged the transition metal dichalcogenide molybdenum disulphide with similar success.

#### 2.1.3 Polarisation-contrast imaging

Many microscopic structures important to biology and biochemistry benefit from polarisation-contrast imaging [24–26]. Anisotropic or chiral structures can rotate the polarisation vector of light propagating through them. The OFM can be used for polarisation-contrast imaging by placing a one linear polariser between the illumination and the sample, and an orthogonal polariser between the tube lens and the sensor. Light that passes through the sample unchanged is extinguished, thus only chiral or anisotropic regions of the sample are imaged. Figure 3(c) shows an image of 4-Cyano-4′-pentylbiphenyl (5CB) liquid crystal droplets, obtained in polarisation-contrast mode with a 40× objective (the same as used previously) and 1.3 ms acquisition times. Illumination is provided by a Nichia NSPL500DS white LED, with ≈250 µW power at the sample plane. Edmund Optics Visible Linear Polarizing Laminated Film (Family ID 1912) was used to both polarize the illumination and analyze the image. The sample was prepared by adding a drop of 5CB liquid crystal to the slide, followed by acetone to form a film. The film is imaged without a cover slip, requiring a dry objective. The droplets can be seen clearly in the bright-field transmission illumination image, and the liquid crystal orientation structure can be seen in the cross-polarised image. The isotropic background is extinguished, appearing dark. Within the droplets, dark regions appear when the 5CB is aligned parallel or perpendicular to the illumination polarisation, and bright regions appear as the crystal orientation deviates from this.

#### 2.1.4 Fluorescence imaging

Fluorescence microscopy is one of the most important and powerful methods for investigating a range of biological processes. By illuminating a fluorescent sample with suitable excitation-wavelength light, emitted light with a Stokes shift in wavelength can be observed. The OFM can perform low-cost fluorescence microscopy by inserting a dichroic beam splitter and optical filters within a printed filter cube between the tube lens and the sensor, and illuminating with an LED of the desired excitation wavelength (Fig. 2(b)).

Figure 3(d) shows fluorescence images of unstained Lily of the Valley (*convallaria majalis*) rhizome taken on the OFM. The same 40× dry objective is used to provide a suitable magnification for the *convallaria majalis* sample. Stray light from external sources at the emission wavelength is minimized by covering the top region of the microscope in a compact opaque box. Two excitation wavelengths (480 nm and 525 nm, with corresponding fluorescence filters as shown in the figure) were used.

Illumination at 480 nm is provided by a Cree C503B-BCS LED, with ≈40 µW at the sample plane. Fluorescence at >510 nm (green) is imaged using a long-pass dichroic (Comar Instruments 550 IY 50) and emission filter (Comar Instruments 510 IY 125), with a 33 ms acquisition time. 525 nm illumination is provided by a Broadcom HLMP-CM2H-130DD LED. Fluorescence at >590 nm (red) is imaged using a long-pass dichroic (Comar Instruments 635 IY 125) and emission filter (Comar Instruments 590 GY 50), with a 600 ms acquisition time.

The inclusion of rubber feet enables a level of vibration damping suitable for acquisition times of the order of hundreds of milliseconds, and can be further improved by situating the microscope in a suitably isolated location, for example on an optical table, and away from desks used for active work. On short time scales (seconds), Allan deviation on the order of nanometers has been measured for the flexure stage mechanism [13], representing adequate performance for fluorescence imaging.

In principle, by selecting appropriate filters and LEDs, illumination modules can be constructed for any fluorescence wavelength as long as the emission is within the wavelength sensitivity range of the sensor. We note that in our current setup, the signal to background ratio is much lower for fluorescence at >590 nm (red) compared to >510 nm (green). This could be improved with the inclusion of a suitable excitation filter.

### 2.2 Assessing optical resolution and distortion

We characterise the resolution of the OFM by calculating the point spread function (PSF) from a black-white edge in an image. This method does not require nanofabrication techniques or fluorescence imaging capabilities, and is thus appropriate for resource-limited settings. The edge is provided by a large square from a USAF resolution test target, covering the height of the field of view. A diffused white LED (Nichia NSPL500DS) is imaged onto the sample by a single-lens critical illuminator, with a numerical aperture of ≈0.48. The sharp edge is then imaged using the Raspberry Pi Camera sensor as in previous sections, with achromatic optics to minimize aberrations.

By aligning and averaging several rows of an image with a vertical edge, we recover an edge response function with low noise. Rows are averaged together using a smoothing spline, which provides both noise rejection and sub-pixel alignment. Differentiating this edge response function (after taking the square root to convert from intensity to amplitude) yields a point spread function, with a full width at half maximum of 480nm for the 40×, 0.65NA Plan-corrected objective used in section 2.1. This is very close to diffraction limited performance, over the central 50% of the field of view. Towards the edges of the field of view both field curvature and aberration are visible, resulting in decreased resolution. While this can be alleviated to some extent by acquiring multiple images at different z positions, our preferred solution is to tile images together, such that only the central region of each tile is used in the final image, and the lower-quality edges are only required for alignment.

In this configuration, the field of view is 350 µm × 262 µm, calibrated using a 1951 USAF resolution test chart. At full imaging resolution, this corresponds to a distance-per-pixel of ≈100 nm. Due to the presence of a Bayer filter, a 2×2 block of pixels (≈200 nm) must be smaller than the optical resolution to avoid undersampling the image. At our optical resolution of ≈480 nm, the measured field of view is within this limit, and a significant increase would come close to undersampling. While this field of view is smaller than most smartphone-based microscopes [16], the need for a wider field of view is alleviated by our precise positioning mechanics, enabling whole-slide imaging at high resolution.

By scanning a sample with straight edge across the field of view it is possible to measure distortion in the optical system. As the optics are axisymmetric, we would expect any distortion also to be axisymmetric Thus, a straight line would appear to deform as it moved across the field of view (appearing straight only in the centre). Performing this experiment with a variety of optical configurations (including an RMS, Plan-corrected objective and a spatially offset Raspberry Pi camera lens), suggested that distortion is very low in this system (less than 0.5%). It is possible that even this slight distortion was affected by the non-uniform response of the Raspberry Pi camera module, and so we suggest it is considered an upper bound.

Calibrating the intensity response of the Raspberry Pi camera module is an important consideration in making the OFM usable. The sensor is designed to be used with a short focal length lens, and consequently suffers from reduced efficiency at the edges of the sensor when used with light that is normally incident across the sensor, such as we have in the microscope. By overriding the default “lens shading table” in the Raspberry Pi’s hardware-accelerated image processing pipeline, we can correct this vignetting, at the cost of increased noise at the edges of the image [19].

### 2.3 Automated imaging

#### 2.3.1 Autofocus

As a sample is translated, deviations in the sample thickness, slide angle, and stage position can cause the image to defocus. As such, autofocus is crucial for automated microscopy. The OpenFlexure Microscope’s software includes two image-based autofocus algorithms, capable of automatically bringing a thin, flat sample into sharp focus.

The first makes use of a Laplacian filter, commonly used for edge detection [27,28], as a sharpness metric. Additional details of this method are given in Appendix A.2. The stage is moved sequentially through a set of positions, and at each position an image is captured as a greyscale array, and the sharpness is calculated from this array. While this method is accurate for the vast majority of samples, it is also slow to run on the Raspberry Pi.

The second option is to measure sharpness while moving the stage continuously, by monitoring the storage size of each frame in the MJPEG video stream. Details of this method are given in Appendix A.3. This method completes in 3–4 seconds, an order of magnitude faster than the Laplacian filter. The speed and reliability of these autofocus options allow for large tiled images to be captured on the microscope.

#### 2.3.2 Tile scans

One of the many advantages of digital microscopy over traditional microscopy is the ability to automatically obtain large quantities of data over both space and time. These are clearly demonstrated by tiled scanning and time-lapse imaging. Tiled scanning allows images much larger than the normal field of view to be obtained by automatically moving around the sample, taking images at a series of locations, and then digitally reconstructing a complete image. Like panorama photography, some overlap is required between the images, but in principle arbitrarily large scans may be taken within the stage’s range of travel. Unlike traditional panorama photography however, the extremely short depth-of-field associated with optical microscopy presents challenges with staying in focus across the scan. In order to properly recombine tiled images, every image must be properly focused. Thus, the microscope should be brought back into focus before taking each image. Autofocus accounts for a significant fraction of the time taken to make a tiled scan, so the JPEG-based fast autofocus method improves acquisition time by a factor of 5–10.

**Fig. 4.**
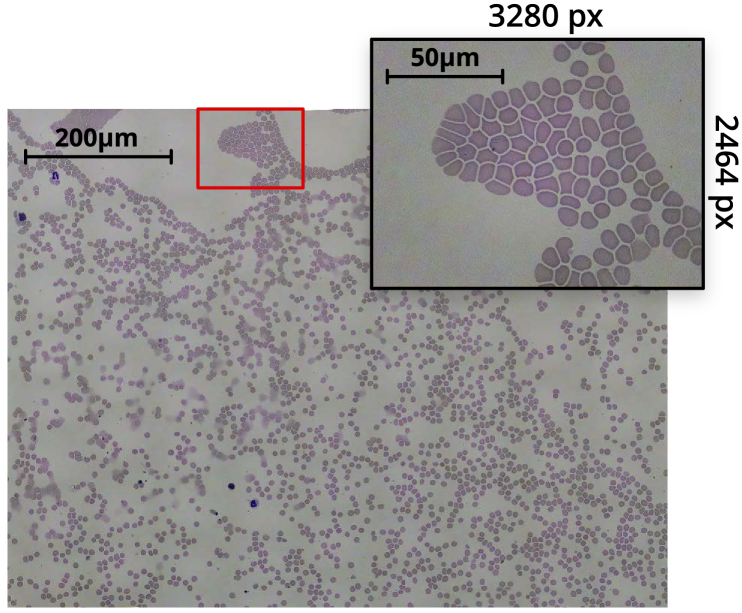
Tiled scan image of a Giemsa-stained thin blood smear, obtained with a 100×, 1.25NA oil immersion objective. The inset highlights an individual 8-megapixel image from the scan. The composite image was obtained from a 10×10 grid of captures. After accounting for image overlap and skewing, and cropping out edges of the composite with missing sections, the resulting image is 14920 px ×11270 px (≈170 megapixel).

Figure 4 shows a stitched tile scan of a blood smear sample, obtained from a 10×10 grid of captures. The composite image was obtained using Image Composite Editor from Microsoft Research [29], taking around 2 minutes to align and stitch the 100 image dataset on mid-range consumer PC hardware. The figure highlights an individual capture, showing the field-of-view of a single image in the scan. At each x-y position, the autofocus routine is performed, before taking a z-stack of 5 images centred on the focus. The central image of each z-stack is used in the stitched image, while the surrounding z-positions are used for different analyses of the sample.

A typical 10×10 grid scan with a 5-image z-stack at each position (500, 8 megapixel images), running a fast autofocus between each x-y position, can be obtained in under 25 minutes. Storing raw bayer data per-image increases the acquisition time. By capturing at lower resolutions, a 10×10×5 scan can complete in around 15 minutes. We have found that depending on where the data is being stored, fluctuations in write-speeds significantly affect acquisition time. However, in future this could be optimised by making better use of parallelisation, reducing the variability in scan acquisition time.

This automation can be used to image samples over long periods of time using time-lapse imaging. Here, the sample position remains fixed, and images are periodically captured. The flexure mechanism is known to drift by tens of microns over the course of several days [13,30], however active correction can be incorporated due to the inclusion of motorized positioning, for example autofocus can be performed before each capture to negate any long-term positional drift in focus. Nevertheless, for long data acquisitions, some level of physical isolation of the microscope is still recommended.

This allows long-term behaviour of samples, for example the growth of biological systems over many days or weeks, to be captured in a data-efficient manner. Slow time-evolution means images need only be captured every few minutes or hours, an impractical and tedious task for a human to perform.

## 3. Software and usability

Usability and user-experience (UX) have been carefully considered to maximise the breadth of functionality available on the OpenFlexure Microscope. While the microscope software stack is designed to be extensible, aided by full documentation and a comprehensive plugin system, non-developers must be considered the primary users. For this reason, the software is split into a server, running on the microscope itself, and client applications which communicate with the microscope over a network. This allows both local control of the microscope via the Raspberry Pi, as well as remote control over a standard Internet Protocol (IP) network. The server software is distributed as a pre-built SD card image for a Raspberry Pi microcomputer [31], and is common to all OpenFlexure Microscopes.

To ensure stability across a wide range of applications, functionality beyond the basics (stage movement, image captures, and basic hardware configuration) is provided by plugins. Developers are able to create new plugins specific to their needs, and enable them on a per-microscope basis. Crucially, plugins can be disabled or entirely removed from the microscope, allowing for a stable environment to be restored whenever required.

Our primary client application, OpenFlexure eV, is a cross-platform graphical application that both exposes basic functionality including a live stream of the microscope camera, and renders user-interfaces for more complex microscope plugins (tile scanning, autofocus, lens-shading calibration, and more) [32]. The application is designed with a strong focus on general usability and UX, especially for non-programmers. Users of the microscope are able to run comprehensive microscopy experiments, with full control over the format and type of data stored, without any programming experience.

## 4. Distributed manufacturing and sustainability

A key aim of the OpenFlexure project is to enable local production. We have demonstrated the local production of microscopes for educational, scientific, and clinical applications at STICLab’s facility in Dar es Salaam, and with our partners at Tech for Trade (Nairobi, Kenya). This has required many optimisations to make the OFM easy to print, assemble, and source parts for, which is equally useful in established research labs. The open-source [33] designs have been replicated in maker spaces and academic labs in numerous countries including in Peru, Germany, Ghana, the USA, and the UK. This replication provides reassurance that the complex printed components can be produced reliably on a wide range of desktop filament deposition printers. This ease of production allows customisation and maintenance of the equipment without external service engineers, and makes the OFM a useful prototyping tool in the development of novel microscopes, or a component to embed within larger instruments.

The 3D printed translation mechanism requires no assembly or alignment. This reduces the bill of materials and speeds up assembly of the microscope. Unlike sliding mechanisms that rely on bearings or smooth ground surfaces, a monolithic flexure mechanism is largely unaffected by dusty or humid environments. The glass transition temperature of PLA (suggested as a print material) is above any reasonable expected operating temperature, at ≈60°C, and while the plastic may deform under heavy loads, the microscope is designed to place only light objects (e.g. a sample, or the optics module) on the moving stages.

Layer-by-layer 3D printing of complicated geometries often requires additional supports structures to be printed, which must be removed by complicated post-print processing. The OFM has been engineered to print without support material, for example by carefully building up unsupported structures using bridges between parts that are present on lower layers, and using a small number of thin “ties” that can be easily and quickly removed during assembly. This makes the design both easier and faster to print, and avoids the risk of damaging printed parts while removing support.

Non-printed parts have been carefully considered to balance cost, performance, and ease of sourcing. Components in the mechanism such as nuts, O-rings, and lenses, push-fit into the design using 3D printed tools. These tools greatly simplify and speed up assembly, allowing each actuator to be assembled in under five minutes. The microscope takes 1–2 hours for an experienced builder to assemble, and typically twice that for a first-time build. Push-fit lens holders ensure that lenses are self-centred, and make it possible to have a single tube extending from the objective to the camera sensor. This sealed tube excludes dust and fungus – a feature introduced in response to trials in The Gambia.

Some components have been carefully chosen to improve the microscope’s lifetime. Previously-used rubber bands perished after weeks to months (regardless of use), and low quality steel screws wore down after 24 hours of continuous use. With quality stainless screws, lubrication, and Viton O-rings, we are able to run microscopes through around 30,000 cycles of stage motion over several months without failure. Some earlier failures were observed in poorly printed microscopes, or microscopes printed using low quality filament. Nevertheless, in these situations another advantage of 3D printed mechanics comes into play: broken parts can be quickly and easily printed locally and replaced with minimal downtime.

Ultimately, the long-term sustainability of a project such as this one depends on the formation of a community, which is now active on the project’s repositories on GitLab.com. As well as questions and bug reports, we have had contributions with fixes and improvements, for example an adapter to connect the OFM to the “UC2” modular optics system [34], or a rugged enclosure developed by STICLab to protect the microscope in transport and use.

## 5. Conclusions

We have demonstrated that the OpenFlexure Microscope design provides a viable platform for research-laboratory microscopy imaging, owing to both compatibility with high-quality optical components, and robust translation mechanics. The OpenFlexure Microscope design provides precise mechanical sample manipulation in a lightweight and compact device, with comparatively trivial assembly. A range of interchangeable imaging configurations and contrast modes have been developed, suitable for a wide range of applications. We have demonstrated bright-field imaging with both epi- and trans- illumination, as well as polarised light microscopy and multi-channel fluorescence imaging using the OpenFlexure Microscope.

Finally, we have demonstrated the high-speed, automated acquisition of thin blood smears for malaria diagnosis. Microscopes produced in Tanzania have been used in the Ifakara Health Institute to obtain large tile scans of patient sample slides. Ring-form trophozoites of *Plasmodium falciparum* are clearly resolved by the microscope, demonstrating that the quality of images are suitable for diagnosis of malaria in-line with the current “gold-standard”. By combining automated image acquisition with suitable machine-learning, rapid, parallel infection diagnosis is possible, significantly increasing the potential throughput of patients in local clinics.

## A. Appendix

### A.1. Estimated cost of production

Costs of common hardware (see Table 1) were obtained from UK retailers, with prices in GBP. For the cost summaries given below, these have been converted to USD at a representative conversion rate of 1.30 USD per GBP.

**Table 1.** Common fastening hardware. Bulk total (using unit prices) 3.80 GBP. One-off total (using pack prices) 19.20 GBP

Name	Qty	Supplier	Code	Pack size	Unit price	Pack price

Common hardware						
M3x25mm hexagon head screws	3	Westfield Fasteners	WF10920	30	0.104 GBP	3.12 GBP
M3 brass nut	4	Westfield Fasteners	WF20152	50	0.049 GBP	2.45 GBP
M3 stainless steel washer	8	Westfield Fasteners	WF10888	200	0.009 GBP	1.8 GBP
M3x8mm cap head screw	14	Westfield Fasteners	WF2344	80	0.035 GBP	2.8 GBP
M2x6mm cap head screws	4	Westfield Fasteners	WF14259	30	0.11 GBP	3.3 GBP
30×2mm Viton Rubber O-Rings	3	Simply Bearings		5	0.56 GBP	2.8 GBP
**Common electronics**						

5mm white LED	1	Farnell	2419079	1	0.23 GBP	0.23 GBP
60 Ohm resistor	1	Farnell	2401722	10	0.0298 GBP	0.298 GBP
**Motorised version**						

M4x6mm button head screws	6	Westfield Fasteners	WF2233	40	0.06 GBP	2.4 GBP

These estimates do not include time of assembly, which is generally 3-4 hours for an inexperienced builder or 1-2 hours for a more experienced builder, per microscope. Likewise, the cost of 3D printers themselves are excluded.

#### Low-cost version

No motors, cheap webcam as sensor and optics, no microcomputer.

• **PLA:** ≈$5 USD.
• **Low-cost webcam:** ≈$5 USD.
• **Common hardware:** Bulk ≈$5 USD, one-off ≈$26 USD (see Table 1)

#### Basic version

No motors, Raspberry Pi microcomputer, and Raspberry Pi camera as sensor and optics.

• **PLA:** ≈$5 USD.
• **Raspberry Pi microcomputer:** $35 USD.
• **Raspberry Pi Camera rev 2.1:** ≈$25 USD.
• **Common hardware:** Bulk ≈$5 USD, one-off ≈$26 USD (see Table 1)

#### High-resolution, motorised version

Motorised, Raspberry Pi microcomputer, Raspberry Pi camera as sensor, RMS objective and tube lens.

• **PLA:** ≈$5 USD.
• **Raspberry Pi microcomputer:** $35 USD.
• **Raspberry Pi Camera rev 2.1:** ≈$25 USD.
• **Common hardware:** Bulk ≈$5 USD, one-off ≈$26 USD (see Table 1)
• 3×**28BYJ-48 micro stepper motors:** ≈$5 USD
• **Achromatic tube lens:** ≈$55 USD.
• **RMS microscope objective:** ≈$50 USD.
• **Motor driver board:** Varies depending on model. Custom board used in this paper ≈$50 USD.

### A.2. General autofocus method

The objective is sequentially moved to a range of z-axis displacements from the current position. The range, and step size, of this sequence can be freely controlled. At each position, an image is captured as an RGB array, and converted into a greyscale image by taking the mean of the three colour channels at each pixel. A 2D Laplacian filter is applied to the image, highlighting regions of spatially rapid intensity changes (indicative of a well-focused image). The image is squared to prevent regions of positive and negative curvature cancelling out, and the sum of all pixels in the filtered image is then used as a sharpness metric. The objective is returned to the position corresponding to the highest sharpness determined by this metric. While this algorithm generally performs well, capturing RGB arrays and filtering each image in sequence is time consuming.

### A.3. Fast autofocus method

For remote control, the microscope’s software serves a real-time MJPEG stream of the camera feed. In this stream, each frame is individually JPEG-compressed. This JPEG compression makes use of the discrete cosine transform. The image is split into blocks, and each block’s data can be described as the superposition of 2-dimensional cosine functions of varying amplitude and spatial frequency. Crucially, sharp image features are associated with high-frequency cosine functions. The JPEG compression algorithm will only store amplitudes for the lowest set of frequencies required to accurately represent the image. Therefore, an image containing sharp features across many blocks will require more stored amplitude values, resulting in a larger output file. For this reason, the size of a JPEG image can, with a few key exceptions, be used as a reasonably good metric for overall image sharpness.

For extremely sparse samples, especially when imaged with a contrast mode resulting in a black background, this metric can fail. Far out of focus, more blocks within the image will contain some information. In focus, very few blocks within the image will contain lots of data (high-frequency amplitudes). In extreme cases, the first effect will become dominant and result in a larger overall file size. For a majority of situations studied so far however, this sharpness metric functions well.

The objective first moves to the top of the scan range, then moves down the full scan range in a single continuous movement. Throughout the movement, the position is recorded against time. Concurrently, the file size of each image in the MJPEG stream is stored against time. These two data sets are the correlated to determine an approximate objective position corresponding to maximum JPEG size. The objective is moved back to this position, and the JPEG size measured again. This value is then compared against the curve obtained in the first step to estimate how much further it needs to go, compensating for mechanical backlash. This final movement brings the objective to its target position.
